# *MitoVariome*: a variome database of human mitochondrial DNA

**DOI:** 10.1186/1471-2164-10-S3-S12

**Published:** 2009-12-03

**Authors:** Yong Seok Lee, Woo-Yeon Kim, Mihyun Ji, Ji Han Kim, Jong Bhak

**Affiliations:** 1 Korean Bioinformation Center (KOBIC), KRIBB, Daejeon 305-806, Korea

## Abstract

**Background:**

Mitochondrial sequence variation provides critical information for studying human evolution and variation. Mitochondrial DNA provides information on the origin of humans, and plays a substantial role in forensics, degenerative diseases, cancers, and aging process. Typically, human mitochondrial DNA has various features such as HVSI, HVSII, single-nucleotide polymorphism (SNP), restriction enzyme sites, and short tandem repeat (STR).

**Results:**

We present a variome database (MitoVariome) of human mitochondrial DNA sequences. Queries against MitoVariome can be made using accession numbers or haplogroup/continent. Query results are presented not only in text but also in HTML tables to report extensive mitochondrial sequence variation information. The variation information includes repeat pattern, restriction enzyme site polymorphism, short tandem repeat, disease information as well as single nucleotide polymorphism. It also provides a graphical interface as Gbrowse displaying all variations at a glance. The web interface also provides the tool for assigning haplogroup based on the haplogroup-diagnostic system with complete human mitochondrial SNP position list and for retrieving sequences that users query against by using accession numbers.

**Conclusion:**

MitoVariome is a freely accessible web application and database that enables human mitochondrial genome researchers to study genetic variation in mitochondrial genome with textual and graphical views accompanied by assignment function of haplogrouping if users submit their own data. Hence, the MitoVariome containing many kinds of variation features in the human mitochondrial genome will be useful for understanding mitochondrial variations of each individual, haplogroup, or geographical location to elucidate the history of human evolution.

## Background

Human mitochondrial genome is 16,569 base pairs (bp) in length, a closed, circular molecule located within the cytoplasmic mitochondria [1]. It has 37 genes: 22 transfer RNA genes, 13 protein coding genes, two ribosomal RNA genes, and one non-coding region containing the origin of replication. Twenty-eight of these genes are encoded by the heavy strand, and nine by the light strand.

Mitochondrial DNA is known for maternal clonal inheritance, rapid evolutionary rate, lack of introns, absence of recombination events, and haploidy [2-4]. Mitochondrial variations are linked to the origin of humans, and play a substantial role in forensics, degenerative diseases, cancers, and the aging process [5]. These mitochondrial DNA roles caused the development of various mitochondrial databases, i.e. MITOMAP containing information related to human evolution, disease [6], mtDB having the human mitochondrial DNAs which have not been deposited in a publicly available database such as GenBank [7], Mitome containing data on whole mitochondrial genomes for metazoan species [8], and MitoRes containing data on nuclear-encoded mitochondrial-related products for metazoan species. However, these databases haven't provided the integrated variation information. The environment of these web-based databases typically lacks a user-friendly and interactive platform for presenting all kinds of variation information.

Here we present an extensive web-based database, MitoVariome. The database will help researchers to identify mitochondrial DNA sequence variation, which is useful for understanding human evolution. It has integrated information such as gene and disease annotation. It provides a user-friendly interface to accept user-defined rules and display graphical views of variations and gene structures.

## Methods and results

**Figure 1:**
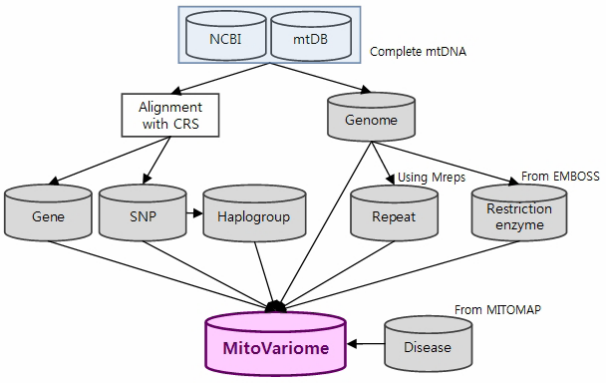
**Flowchart of MitoVariome database construction**.

The MitoVariome database was described below (Figure 1).

### Data source

The database consists of over 5,000 complete human sequences of mitochondrial DNA. They were obtained from the NCBI GenBank database, MITOMAP at http://www.mitomap.org, and mtDB at http://www.genpat.uu.se/mtDB/. The nucleotide position and gene annotation corresponding to those of rCRS (revised Cambridge Reference Sequence) [9] were used. Geographic origin and disease information of human mitochondrial DNA were parsed from the above mentioned databases.

### Assignment of haplogroup

Each human mitochondrial DNA sequence was aligned with the rCRS by ClustalW software and haplogroups were assigned defined by sequence differences from the rCRS. The haplogroup-diagnostic nucleotide sequence variants were assembled from the information from the references [10,11]. The distribution of each haplogroup is shown in statistics page http://variome.kobic.re.kr/MitoVariome/statistics.jsp. The mitochondrial haplogroups in this study are divided into twenty groups broadly. We defined mitochondrial DNA sequences sharing over 95 percent of SNPs of each haplogroup as each haplogroup.

### Finding of short tandem repeats, restriction enzyme sites

We used mreps [12] tool to detect short tandem repeats in mitochondrial DNA. We found all the mitochondrial tandem repeats which have at least two bases in a repeated unit and two copies as the minimum threshold. From the tandem repeat analysis, we found that each human mitochondrial DNA had on average 929 tandem repeat regions. Also, around 31.2% of each mitochondrial genome was detected as tandem repeat regions.

In order to predict restriction enzyme cleavage sites in human mitochondrial DNA, we used the 'restrict' program from the EMBOSS package. These analyses used 19 restriction enzymes which are frequently used in several studies on mitochondrial genomes. Each human mitochondrial DNA had on average 506 restriction enzyme sites http://variome.kobic.re.kr/MitoVariome/view_enzymelist.jsp. On average there were 99 MseI restriction enzyme sites (TTAA) at the most and one XhoI restriction enzyme site (CTCGAG) at the least.

### Mapping disease information

Mapping disease information to mitochondrial DNA was performed by using mitochondrial disease information (Leber Hereditary Optic Neuropathy, Alzeimer's Disease, Parkinsons's Disease, and so on) on coding and non-coding regions. This information was obtained from Pubmed and MITOMAP. There are forty-two reported mitochondrial DNA base substitution diseases in our database (disease listing in http://variome.kobic.re.kr/MitoVariome/view_diseaselist.jsp, and 235 sites were related to base substitution diseases.

### Visualization of results

Tables and two-dimensional view are provided to the users as visualize the final resultants. The tables provide detailed information of genome/gene and diseases associated with variation data. This information linked to GenBank and Pubmed databases with ID number and users can download sequences in FASTA files. The two-dimensional view result is shown by a method developed using Gbrowse [13]. This browser provides a graphical view of the mitochondrial genome/gene structure and the results obtained from above mentioned analyses.

## Web interface

**Figure 2:**
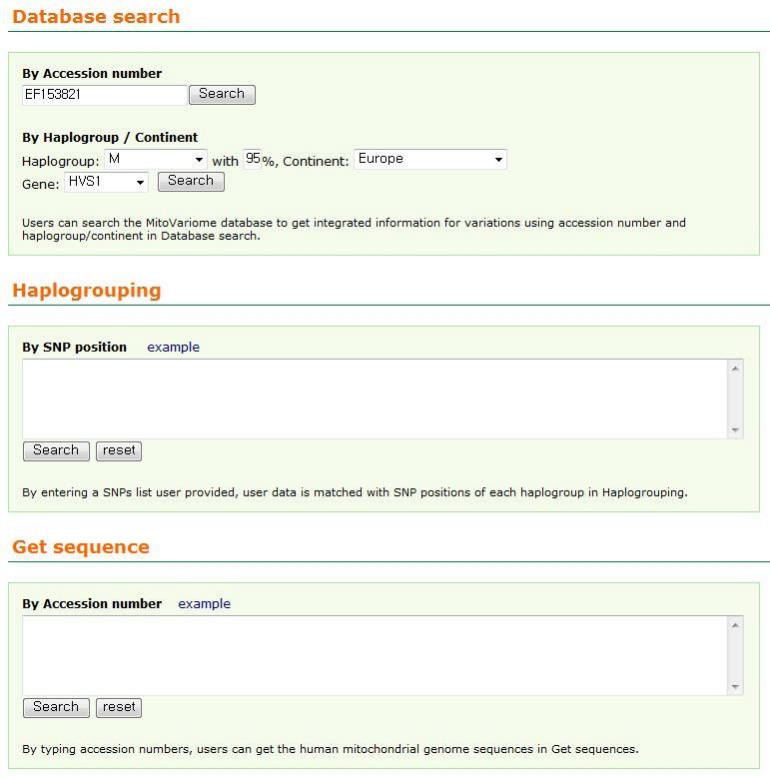
**Search interface of MitoVariome**. There are three functions; *Database search*, *Haplogrouping *and *Get sequences*. Users can search the MitoVariome database to get integrated information for variations using accession number and haplogroup/continent in *Database search*. By entering a SNPs list user provided, user data is matched with SNP positions of each haplogroup in *Haplogrouping*. By typing accession numbers, users can get the human mitochondrial genome sequences in *Get sequences*.

MitoVariome provides a web interface that allows researchers to browse the integrated information of mitochondrial DNA variation and disease. As shown in Figure 2, users can search using two entries; accession number (from NCBI), and haplogroup/continent. By submitting an accession number or haplogroup/continent information with haplogroup matching percent optionally (default 95%), the user can obtain genome or gene information related to the query and information for variations within the queried genome. The summary shows geographic information, haplogroup, sequence, reference and the number of SNP, disease related SNP, short tandem repeat, SNP within short tandem repeat, restriction enzyme site, and SNP within restriction enzyme site. MitoVariome also provides detailed disease information with associated variations in a table. For an advanced study, this database provides nucleotide sequences in a FASTA format for all the entries.

There are two additional applications in the database for further study, *Haplogrouping *and *Get sequences*. In order to assign haplogroup with user provided data, MitoVariome has the *Haplogrouping *application. By entering a user provided SNPs list, MitoVariome is matching with SNP positions in each haplogroup. It provides a table with over 50% matched haplogroup(s); haplogroup name, matched SNPs, unmatched SNPs, the count of matched SNPs and SNPs in each group, and percentage of matched over SNPs in each group. The second application, *Get sequence*, is provided in MitoVariome. Users can download the human mitochondrial genome sequences by typing accession numbers.

**Figure 3:**
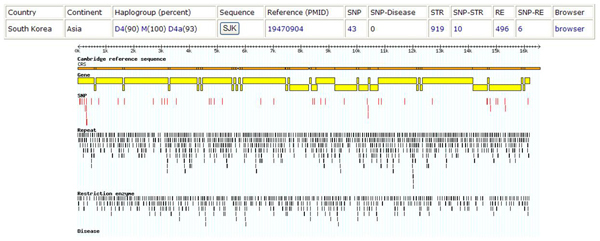
**Example of MitoVariome ('SJK')**. Variation information and a corresponding two-dimensional image are shown. The top box is variation information. The top line with small bars is a scale bar for mitochondrial genome. The light brown horizontal bar is the Cambridge Reference Sequence. The yellow bars and blocks are CDS. The red vertical bars below the yellow bars are SNPs. Small black vertical bars are STRs. The last small black vertical bars are restriction enzyme sites.

Here, we present an example of MitoVariome's usage for studying genetic variations in Korean mitochondrial genome. A Korean genome (SJK) derived mitochondrial genome was used as input for MitoVariome. SJK is the first full length Korean individual genome sequence [14]. We found 43 novel SNPs by comparing SNPs with rCRS. Two insertions and one deletion were detected with six non-synonymous SNPs. STR has 919 tandem repeat regions and 496 restriction enzyme sites showing similar numbers with the average of all human mitochondrial genome. 15 of 44 mtDNA variations are referred as the marker of "D4" haplogroup which is prevalent in Korea. Figure 3 provided variation information and a corresponding two-dimensional image.

## Conclusion

MitoVariome was constructed as a database and analysis server for human mitochondrial DNA variation. It provides a platform to retrieve all kinds of variation information found in human mitochondrial DNA. MitoVariome provides assignment for the haplogroup with user provided SNP list by using rCRS information. MitoVariome database can be accessed at http://variome.kobic.re.kr/MitoVariome.

## Competing interests

The authors declare that they have no competing interests.

## Authors' contributions

YSL, WYK, and MJ wrote the code for MitoVariome. MJ and WYK worked on the update the website and designed the database. WYK designed the overall web site. JHK wrote the code for graphical results. YSL wrote the main draft of the paper and coordinated the project. JB directed the whole variome project in KOBIC including MitoVariome and supervised the manuscript. All authors have read and approved the final manuscript.

## Note

Other papers from the meeting have been published as part of *BMC Bioinformatics *Volume 10 Supplement 15, 2009: Eighth International Conference on Bioinformatics (InCoB2009): Bioinformatics, available online at http://www.biomedcentral.com/1471-2105/10?issue=S15.
